# Progress in Surgery

**Published:** 1895-10-12

**Authors:** 


### PROGRESS IN SURGERY.

### SURGERY OF THE LIVER AND GALL BLADDER.

**Cholelithiasis**, according to Jordan Lloyd,[1] is one of the most frequently overlooked disorders. According to this writer, the situations in which gall stones may be found may be divided into two groups—first, those in which the walls of the gall bladder are yielding, and allow of its distension; and, secondly, when the walls are contracted, indurated, and indistensible. In the first group the stones may be in the gall bladder, in the cystic duct, or in the common bile duct. In the second group they lie in the shrunken gall bladder or cystic duct, in the common duct, which, with the hepatic duct, is thickened and dilated, whilst the gall bladder and its duct are shrunken and indurated; or the gall bladder is packed with stones, and a calculus is also present in the common duct. When the stone is in the gall bladder there is usually no tumour, no jaundice, and no pyrexia, unless a stone passes along the ducts to the duodenum. For this condition Lloyd regards cholecystostomy as the proper operation, and that cholecystectomy and cholecystenterostomy are unjustifiable. A stone in the cystic duct is marked by colic and the presence of a tumour formed by the distended gall bladder. There is no jaundice. Cholecystostomy is the operation which should first be tried, but may be supplemented if necessary by cholelithotrity or choledochotomy if the stone cannot be removed after the gall bladder has been incised. Should these procedures fail we may leave the stone where it is, complete our cholecystostomy, and endeavour to remove the obstruction by daily injections of oil or water, or we may perform cholecystenterostomy, which is more justifiable here than in the condition first described. If the stone is in the common bile duct we get symptoms of colic, tumour, jaundice, and sometimes pyrexia. The operative treatment is practically the same as when the stone is in the cystic duct, but is more difficult of performance, owing to the greater depth of the stone. It is well to remember that chronic jaundice, accompanied by a distended gall bladder, is most commonly due to malignant disease of the liver or pancreas, and is unaccompanied by pyrexia. When the stone lies in the shrunken gall bladder or cystic duct diagnosis is most difficult, but may be suspected where paroxysms of pain are frequently recurring, and where pyrexia and local tenderness are present. There is no tumour and no jaundice. The gall bladder is so contracted that cholecystostomy and cholecystenterostomy are impossible. Cholecystendysis is possible, but very difficult and dangerous. Cholecystectomy is available and in some circumstances should be tried. It is by choledochotomy, therefore, or cholecystotomy without suture, that we can most efficiently deal with this class of case, and it is upon free and perfect peritoneal drainage by means of glass tubes and iodoform tampons that we have to rely. When there is a stone in the common bile duct with a shrunken gall bladder, or with a gall bladder full of stones, we get the symptoms which Osler has described as characteristic of chronic obstruction of the common bile duct with gall stones, viz., (1) paroxysmal hepatic colic; (2) well-marked ague-like attacks, characterised by chill, sweating, and fever; (3) chronic jaundice of varying intensity, deepening after each paroxysm, and fading during the intervals. The only operations available for these conditions are cholelithotrity and choledochotomy, and Lloyd favours the latter without any attempt at suturing the duct. A separate incision may be made into the gall bladder, if it also contains stones. Waring[2] thinks the following signs and symptoms point to the necessity of surgical interference in cases of cholelithiasis: (1) The presence of a tumour in the abdomen which appears to be a large and distended gall bladder. (2) Jaundice which is persistent, together with other signs and symptoms which point to complete obstruction of the common hepatic or common bile duct. (3) The occurrence of successive paroxysmal attacks of biliary colic, with short intervals between, lowering the general health, and not amenable to medical measures. (4) Symptoms of localised inflammation in the region of the gall bladder, associated with attacks of biliary colic.

(5) The occurrence of acute peritonitis, probably the result of perforation of the gall bladder or bile ducts. Pridgin Teale[3] thinks that the use of the needle to break up impacted biliary calculi may prove to be an important substitute for the crushing forceps. Where the gall stone will yield to the crushing effects of the thumb and forefinger, this is probably the best and safest method. But when this fails, he thinks the needle passed through the walls of the duct will prove to be more certain and less dangerous than either the use of crushing forceps or removal of the calculus by incision of the duct. Hume[4] thinks that when the peritoneal cavity is opened transversely the neighbourhood of the gall bladder and bile ducts is much more accessible both to the eye and the hand. A further advantage of the transverse incision is efficiency in drainage. Hellier[5] records a case of cholecystotomy in which the condition which Riedel calls "linguiform appendix of the liver" was present. This hypertrophic condition not infrequently accompanies cholelithiasis, but often, as in the case referred to, leads to difficulties of diagnosis. Block[6] advocates a new method of cholecystotomy which he has employed successfully in one case. In this method, called extra-abdominal cholecystotomy, the distended gall bladder is drawn through the abdominal wound, and a portion about the size of a hen's egg is fastened outside this wound by sutures until adhesions have formed and the peritoneal cavity has been closed. The gall bladder is then opened, and the stones removed. The wound in its coats having been closed by sutures, the gall bladder, after a further interval to allow of complete cicatrisation, is separated from the margins of the incision in the abdominal wall, and returned into the peritoneal cavity, the external wound being finally closed. Kehr[7] reports that of seventy operations for cholelithiasis he has met with incarcerated concretions in the cystic duct in twenty-six cases. The ordinary operation not resulting satisfactorily in seven cases, he adopted the plan of direct excision of the stone from the duct, and sewing up the opening through which it was removed. Tuffier[8] has proposed a lumbar choledochotomy for those cases in which the lesion is in the common bile duct. He says that the common bile duct, the second portion of the duodenum, and the posterior surface of the pancreas lie outside the peritoneum, and the common bile duct can therefore be exposed by the following procedure: The patient is placed on his left side, a pillow having been placed underneath the loin. An incision is made parallel to the twelfth rib, and one finger's breadth below its lower border, commencing at the angle and passing inwards for about 15 cm. The lower border of the kidney is defined and pushed upwards with a retractor. The inferior vena cava is raised and pushed inwards, and the second portion of the duodenum, along with the head of the pancreas, is drawn outwards. By means of the finger a cord can now be felt at the bottom of the wound, which passes upwards, and consists of the common bile duct and its accompanying vessels. By careful dissection, that part of the common bile duct which lies in the pancreas and behind the duodenum can be isolated and examined. Shepherd[9] records a case of cholecystenterostomy performed with Murphy's button which died on the fourth day from hæmorrhage. It proved to be a case of malignant disease of the head of the pancreas, and was, therefore, an unsuitable case for the use of the button. Swain[10] records a successful case of cholecystenterostomy with Murphy's button, and Abbe[11] a case in which, ten months after operation by Murphy's button, the opening between the gall bladder and duodenum had become absolutely stenosed by cicatricial contraction. Morris,[12] in discussing the clinical confusion between distension of the gall bladder and movable kidney thinks the following points should be considered in our attempts at establishing a diagnosis: (1) The enlarged gall bladder as well as the kidney is a frequent cause of movable abdominal tumour. (2) Always inquire if there has been a distinct attack of jaundice. (3) The tumour caused by an enlarged gall bladder can in almost all cases be invariably felt, whereas a movable kidney (unless also enlarged) cannot. The latter is sometimes easily detected, at others not at all. (4) The fact that the size of the tumour varies from time to time goes for nothing in the diagnosis, unless it is clear that with the diminution of the swelling there invariably follows a marked increase in the quantity of urine voided. (5) A gall bladder with calculi feels much harder than a movable kidney. (6) However free the movements of a gall bladder, they take place in the arc of a circle, the centre of which is a point beneath the edge of the right lobe of the liver; but, unless the liver as a whole is unduly mobile, the gall bladder cannot be pushed downwards towards the pelvis though it descends a little on deep inspiration. The kidney, on the other hand, moves bodily from place to place within the limits of its loose connection. The kidney has an especial tendency to slip beneath the fingers into its normal position in the loin, but the gall bladder, though it can, in many cases, be pushed far into the loin, has the tendency to spring back again to its position in the front of the abdomen. (7) Too much stress has been laid, in the symptoms of movable kidney, upon an undue hollowness and resonance, with diminished resistance in the loin, and upon the relationship of the colon to the tumour. (8) Aspiration is a dangerous and uncertain means of diagnosis. (9) In the doubtful cases an exploratory incision should be undertaken as the only means of positively deciding the diagnosis.

**Hydatids of the Liver** have been operated upon successfully in cases recorded by Depage[13] and Malcolm.[14]

**Hepatic Abscess.**—Louguet[15] records an interesting case of this condition which was operated upon three times, and on the third occasion four and a half litres of pus drained away. The remarkable points of the case were: (1) The abscess supervened without cause. (2) Only a few cases are recorded of an equal or greater quantity of pus. (3) The pus was sterile; this fact is well known, this case being the thirty-eighth recorded. There exist several suppositions to account for this sterility: (1) That the microbes are killed by the bile; recent researches have shown this to be untenable. (2) Chauffard's view that the hepatic cell by a chemical action yet unknown causes the pathogenic bacteria to disappear; the fact that sterile pus is frequently met elsewhere (as in the Fallopian tubes) is an objection to this supposition. (3) The theory of late secondary sterility; this finds most supporters; in proportion to the length of duration of the case the greater the chance of finding sterile pus. Curnow,[16] in a case of hepatic abscess followed by amœbic dysentery, has shown the causal connection between dysentery and the so-called tropical abscess of the liver by the discovery of the amœba dysenteriæ in the pus, from the liver, and in the evacuations. He says the migration of the amœba coli from the intestine to the liver must now take the place of chill, excessive eating and drinking, tropical climate, and other vague generalities to which tropical abscess is so frequently ascribed.

**Perforation of the Gall Bladder following Typhoid Fever.**—A case of this rare event is recorded by Monier-Williams and Shield.[17] The patient was subjected to operation, the wound treated by gauze tamponnage, and the patient recovered. The gall-bladder was in a state of acute inflammation at the time of operation, and a small perforating ulcer was found near the neck of the gall-bladder which contained pus.

[1] Birm. Med. Review, April, 1895, p. 195. [2] Clinic. Journ., Jan. 16, 1895, p. 186. [3] Brit. Med. Journ., Feb. 2, 1895, p. 237. [4] Lancet, Feb. 16, 1895. [5] Brit. Med. Journ., May 4, 1895, p. 977. [6] Brit. Med. Journ., March 30. 1895; and Revue de Chir., Feb., 1895. [7] Annals of Surg., Feb., 1895, p. 213. [8] Brit. Med. Journ., June 23, 1895. [9] Annals of Surg., May, 1895, p. 581. [10] Lancet, March 23, 1895, p. 743. [11] Annals of Surg., May, 1895, p. 485. [12] Brit. Med. Journ., Feb. 2, 1895, p. 238. [13] Med. Chron., May, 1895, p. 127. [14] Lancet, June 22, 1895, p. 1,570. [15] Brit. Med. Journ,, April 3, 1895; and La Presse Méd., March 16, 1895. [16] Lancet, May 4, 1895. [17] Lancet, March 2, 1895, p. 534.

